# Supplementary material for: Anion-Sensing Properties of Cyclopentaphenylalanine
Source: Molecules. 2022 Jun 18;27(12):3918. doi: 10.3390/molecules27123918 (PMC9228215; doi:10.3390/molecules27123918)
Supplement: Supplementary file 1 [file molecules-27-03918-s001.zip › molecules-1778532-supplementary.pdf]

Supporting Information

Anion-sensing properties of cyclopentaphenylalanine

MeCN

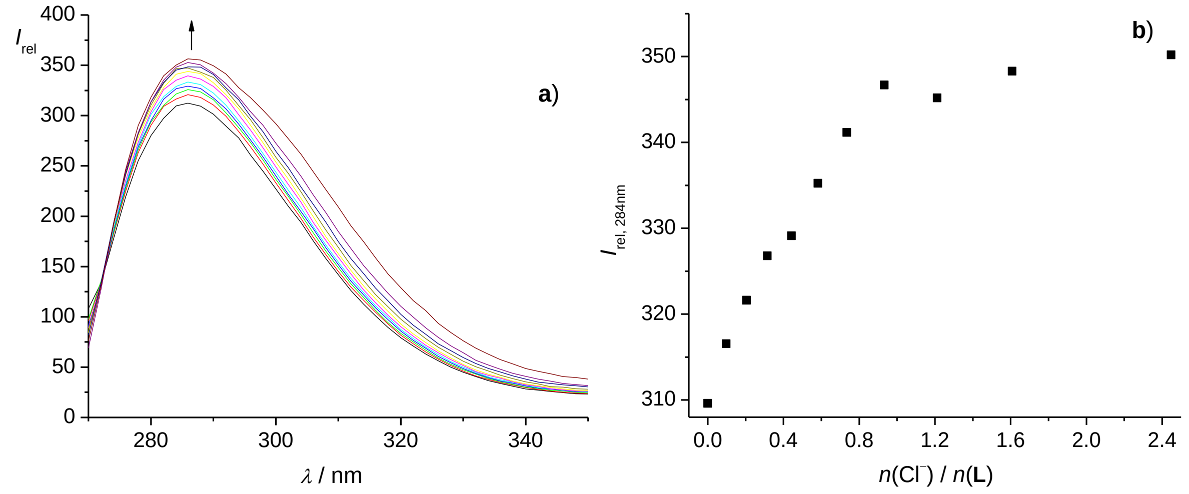

Figure S1. a) Fluorimetric titration of **L** (c = 5.29 × 10^{-5} mol dm^{-3}) with TEACl (c = 2.84 × 10^{-4} mol dm^{-3}) in acetonitrile at 25 °C. λ_{ex} = 260 nm, excitation slit = 10 nm, emission slit = 10 nm. b) Dependence of relative fluorescence intensity on $n(Cl^-)$ / n(**L**) molar ratio.

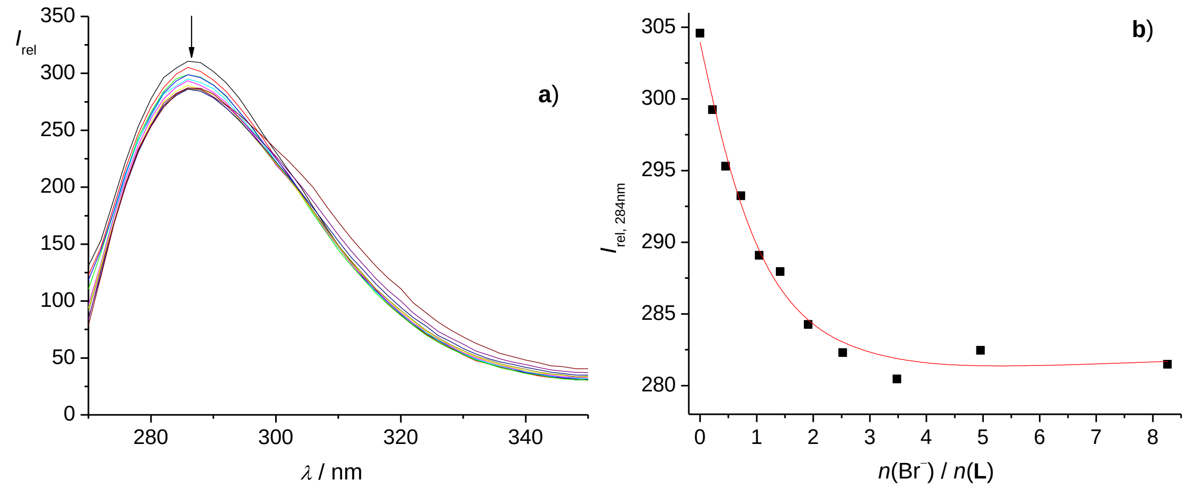

Figure S2. a) Fluorimetric titration of **L** (c = 4.00 × 10^{-5} mol dm^{-3}) with TBABr (c = 8.00 × 10^{-4} mol dm^{-3}) in acetonitrile at 25 °C. λ_{ex} = 260 nm, excitation slit = 10 nm, emission slit = 10 nm. b) Dependence of relative fluorescence intensity on $n(Br^-)$ / n(**L**) molar ratio. ■ experimental; — calculated.

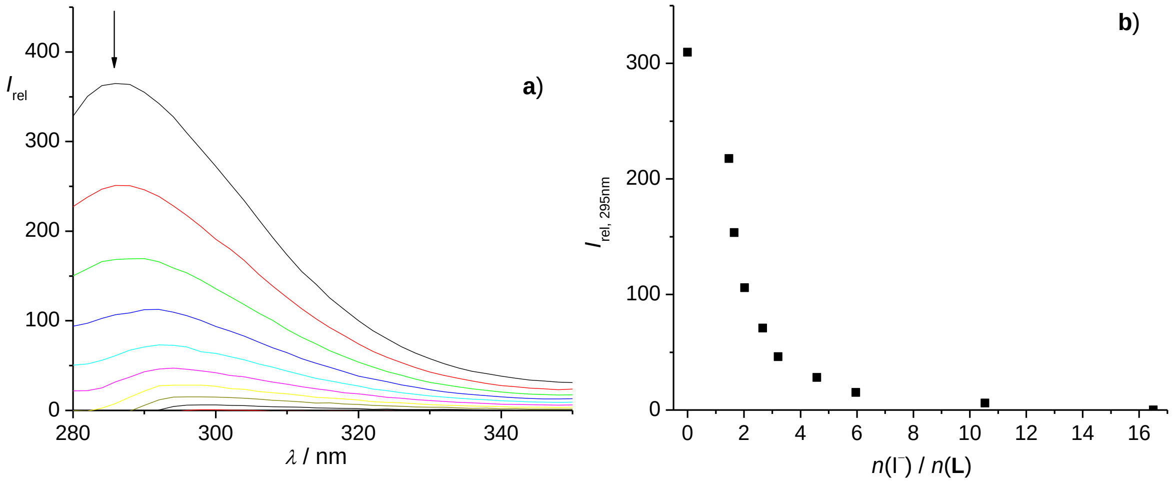

Figure S3. a) Fluorimetric titration of **L** (c = 4.34 × 10^{-5} mol dm^{-3}) with TBAI (c = 9.15 × 10^{-3} mol dm^{-3}) in acetonitrile at 25 °C. λ_{ex} = 260 nm, excitation slit = 10 nm, emission slit = 10 nm. b) Dependence of relative fluorescence intensity on $n(I^{-})$ / n(**L**) molar ratio.

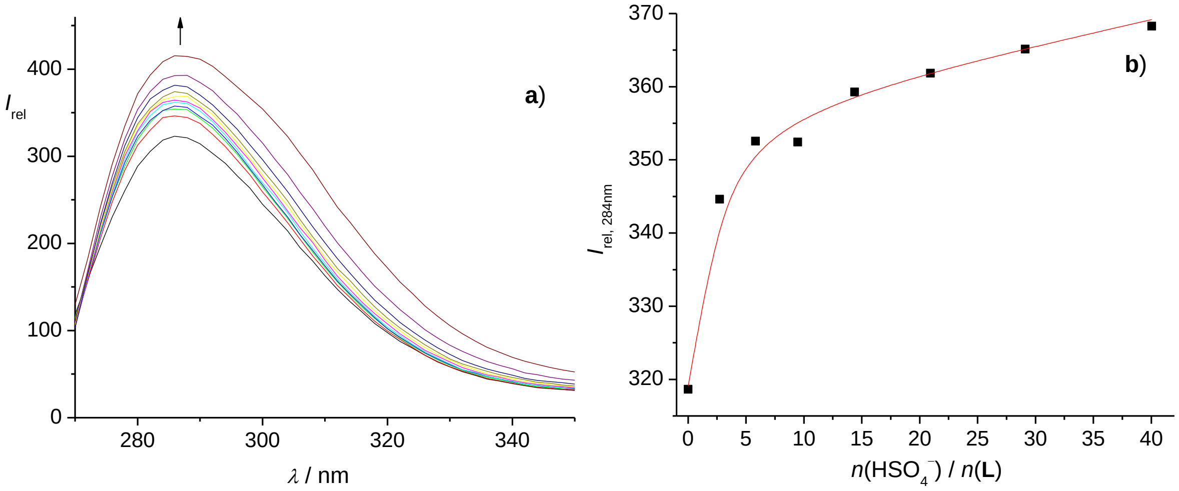

Figure S4. a) Fluorimetric titration of **L** (c = 3.71 × 10^{-5} mol dm^{-3}) with $TBAHSO_4$ (c = 1.55 × 10^{-2} mol dm^{-3}) in acetonitrile at 25 °C. λ_{ex} = 260 nm, excitation slit = 10 nm, emission slit = 10 nm. b) Dependence of relative fluorescence intensity on $n(HSO_4^{-})$ / n(**L**) molar ratio. ■ experimental; — calculated.

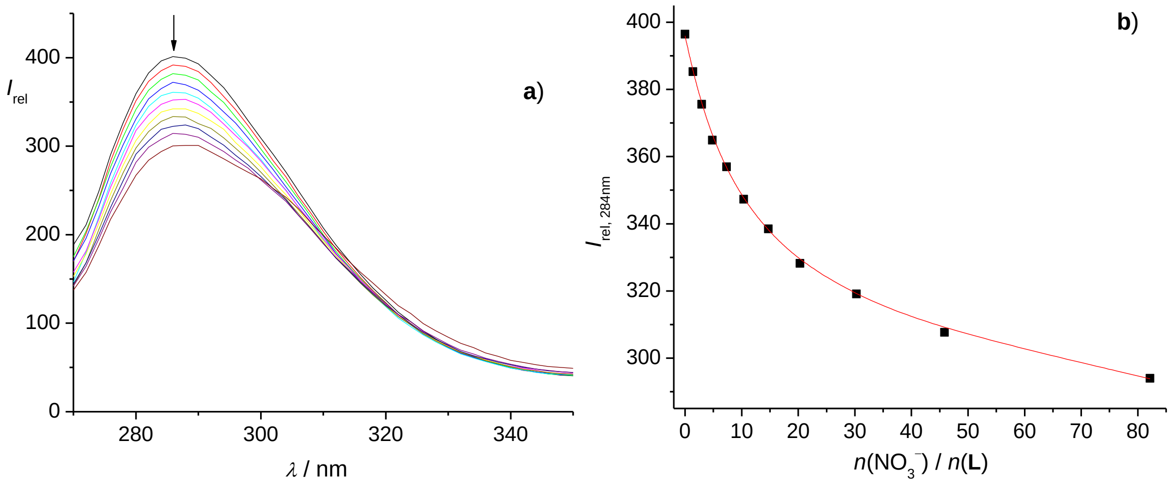

Figure S5. a) Fluorimetric titration of **L** (c = 4.57 × 10^{-5} mol dm^{-3}) with $TBANO_3$ (c = 9.09 × 10^{-3} mol dm^{-3}) in acetonitrile at 25 °C. λ_{ex} = 260 nm, excitation slit = 10 nm, emission slit = 10 nm. b) Dependence of relative fluorescence intensity on $n(NO_3^-)$ / n(**L**) molar ratio. ■ experimental; — calculated.

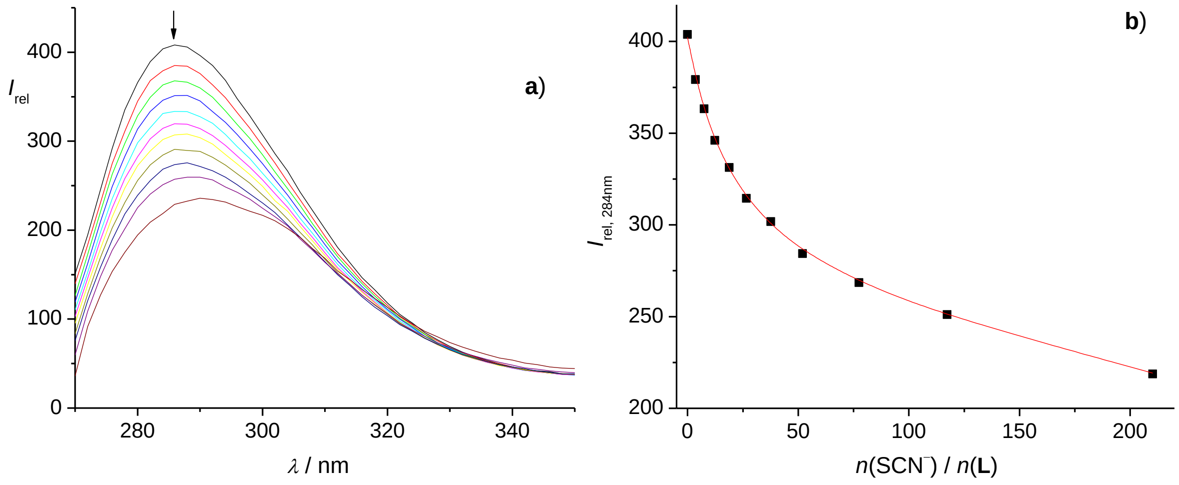

Figure S6. a) Fluorimetric titration of **L** (c = 5.94 × 10^{-5} mol dm^{-3}) with TBASCN (c = 0.0302 mol dm^{-3}) in acetonitrile at 25 °C. λ_{ex} = 260 nm, excitation slit = 10 nm, emission slit = 10 nm. b) Dependence of relative fluorescence intensity on $n(SCN^-)$ / n(**L**) molar ratio. ■ experimental; — calculated.

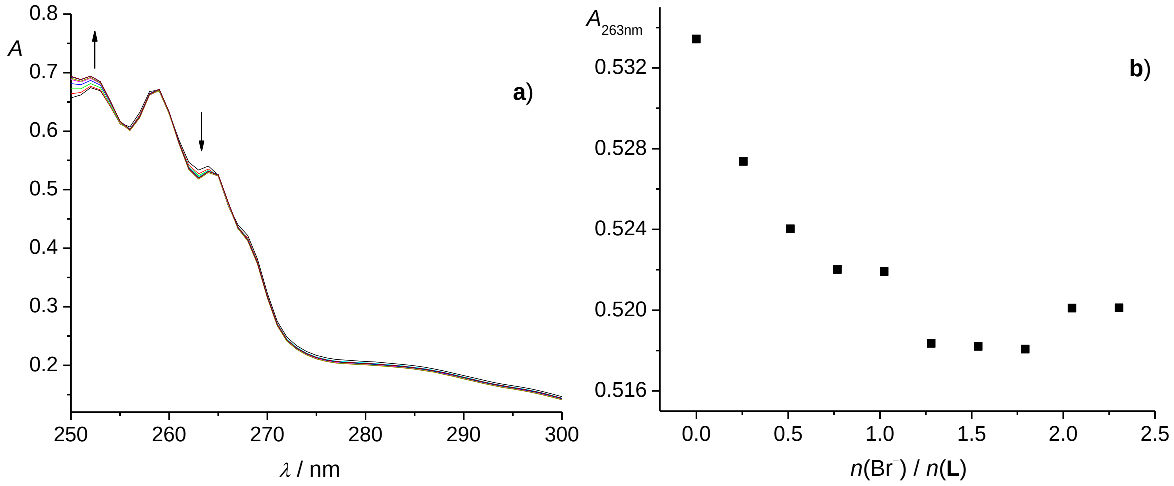

Figure S7. a) Spectrophotometric titration of **L** (c = 4.42 × 10^{-4} mol dm^{-3}) with TBABr (c = 5.03 × 10^{-3} mol dm^{-3}) in acetonitrile at 25 °C. b) Dependence of absorbance on $n(Br^{-})$ / n(**L**) molar ratio.

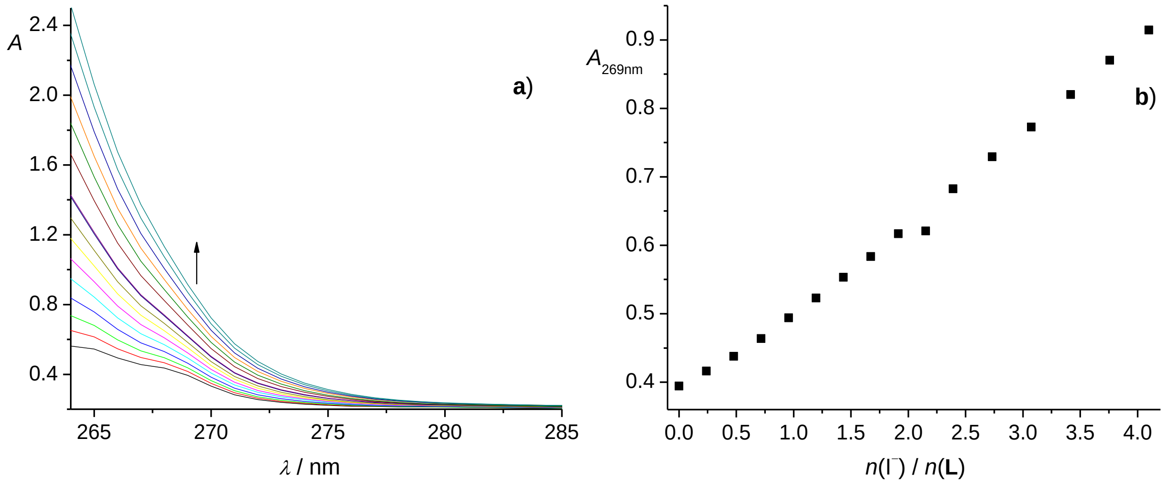

Figure S8. a) Spectrophotometric titration of **L** (c = 4.42 × 10^{-4} mol dm^{-3}) with TBAI (c = 0.0151 mol dm^{-3}) in acetonitrile at 25 °C. b) Dependence of absorbance on $n(I^{-})$ / n(**L**) molar ratio.

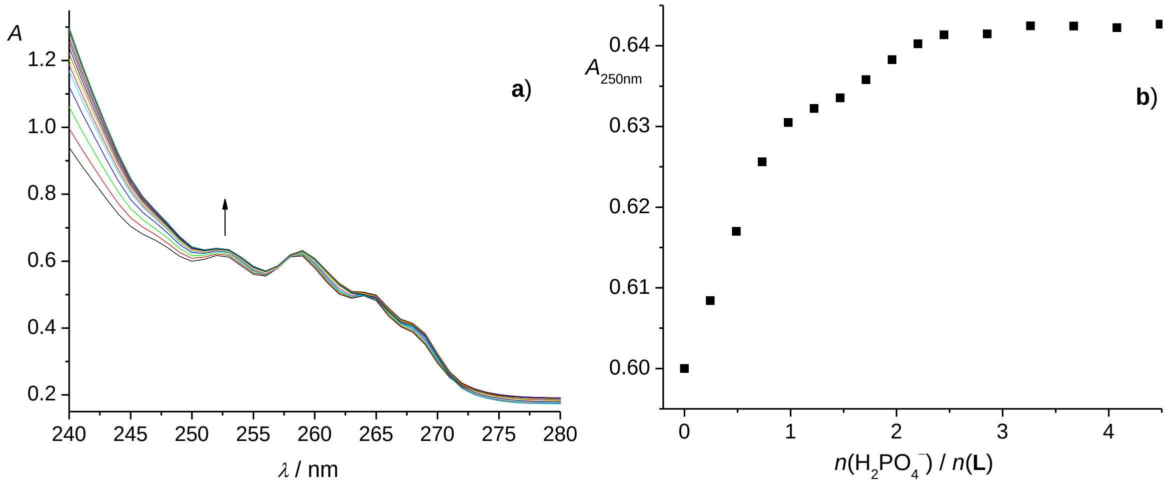

Figure S9. a) Spectrophotometric titration of **L** (c = 4.23 × 10^{-4} mol dm^{-3}) with $TBAH_2PO_4$ (c = 3.45 × 10^{-3} mol dm^{-3}) in acetonitrile at 25 °C. b) Dependence of absorbance on $n(H_2PO_4^-)$ / n(**L**) molar ratio. ■ experimental; — calculated.

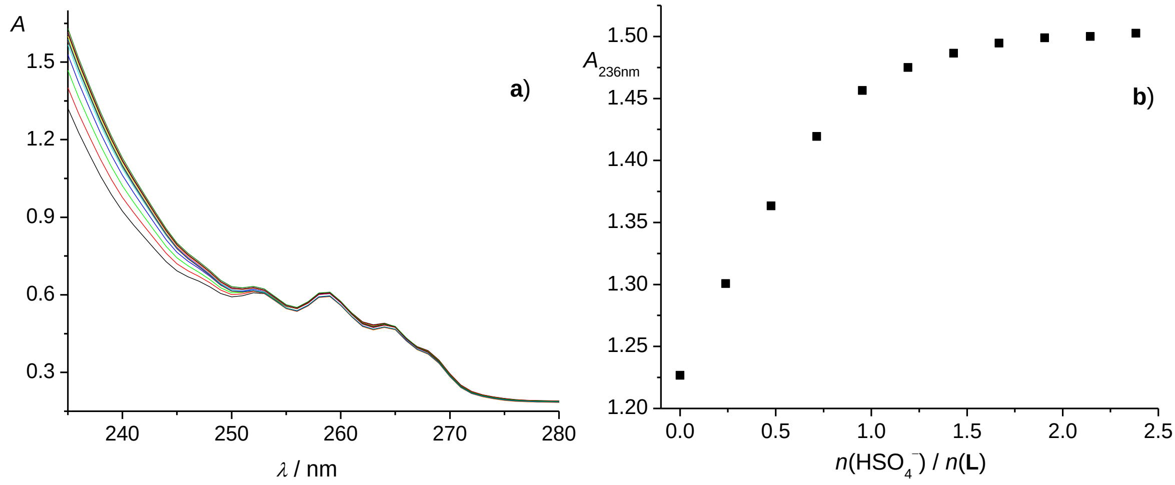

Figure S10. a) Spectrophotometric titration of **L** (c = 4.23 × 10^{-4} mol dm^{-3}) with $TBAHSO_4$ (c = 2.88 × 10^{-3} mol dm^{-3}) in acetonitrile at 25 °C. b) Dependence of absorbance on $n(HSO_4^-)$ / n(**L**) molar ratio. ■ experimental; — calculated.

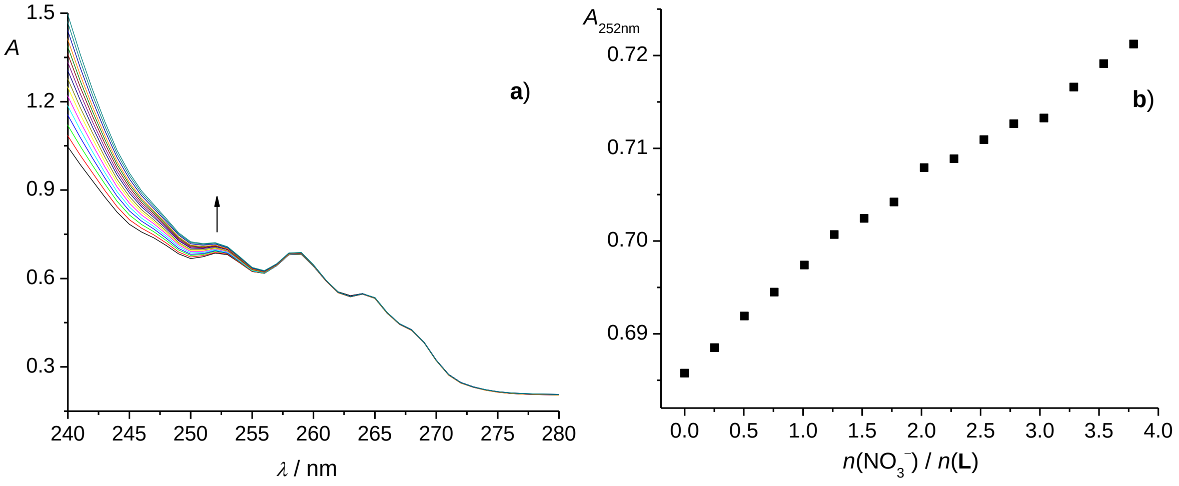

Figure S11. a) Spectrophotometric titration of **L** ($c = 4.42 \times 10^{-4}$ mol dm^{-3}) with $TBANO_3$ ($c = 1.49 \times 10^{-2}$ mol dm^{-3}) in acetonitrile at 25 °C. b) Dependence of absorbance on $n(NO_3^-)$ / n(**L**) molar ratio. ■ experimental; — calculated.

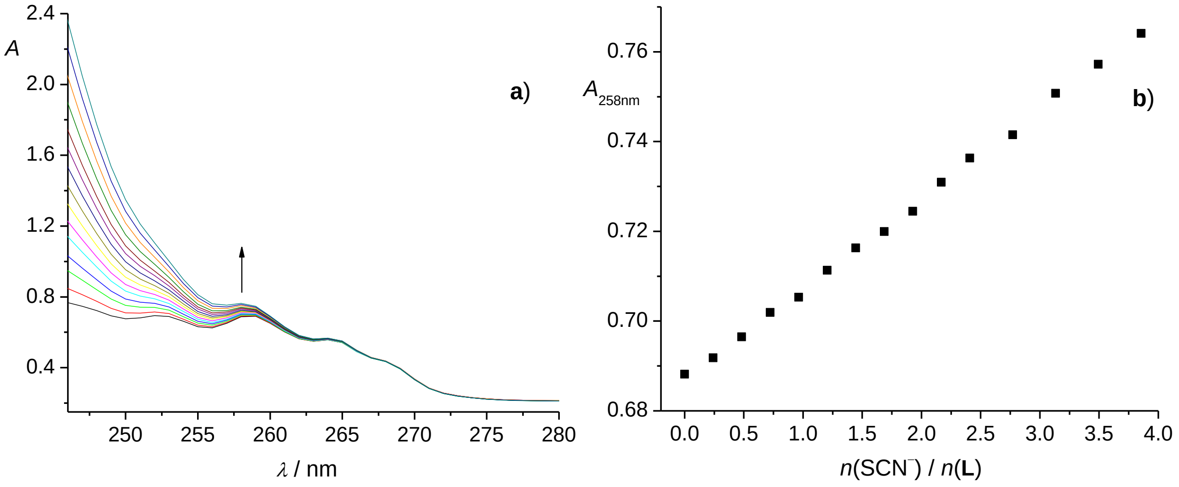

Figure S12. a) Spectrophotometric titration of **L** ($c = 4.42 \times 10^{-4}$ mol dm^{-3}) with TBASCN (c = 0.0213 mol dm^{-3}) in acetonitrile at 25 °C. b) Dependence of absorbance on $n(SCN^-)$ / n(**L**) molar ratio.

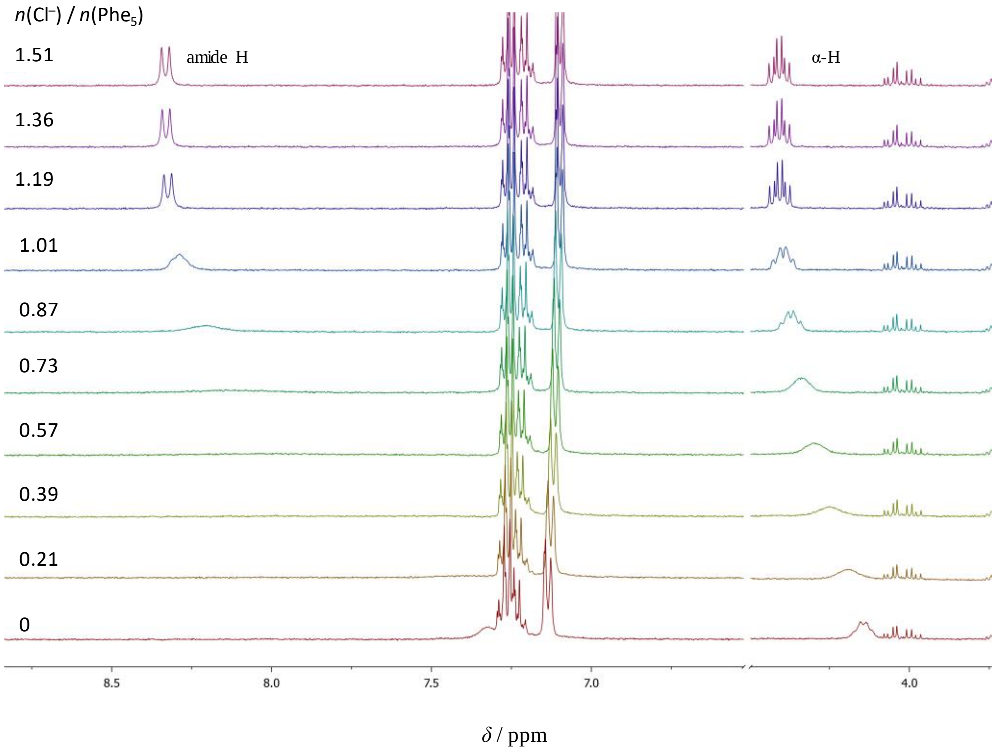

Figure S13. ^{1}H NMR titration of **L** (c = 5.17 × 10^{-4} mol dm^{-3}) with TEACl (c = 2.33 × 10^{-3} mol dm^{-3}) in acetonitrile at 25 °C.

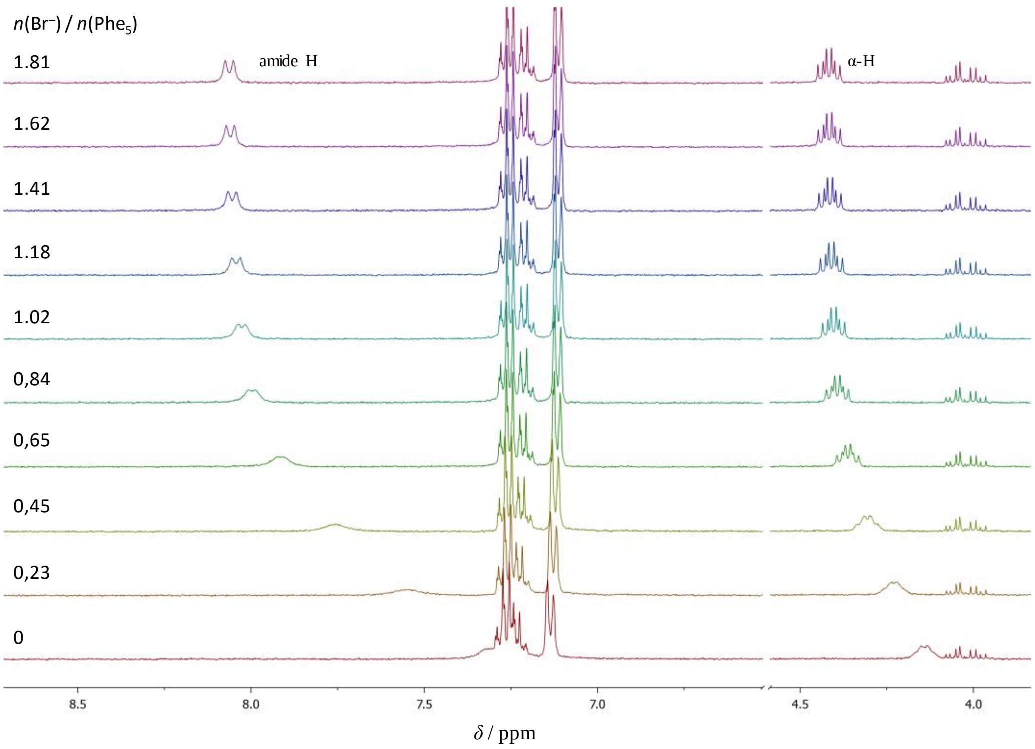

Figure S14. ^{1}H NMR titration of cyclopeptide **L** (c = 4.26 × 10^{-4} mol dm^{-3}) with TBABr (c = 2.61 × 10^{-3} mol dm^{-3}) in deuterated acetonitrile.

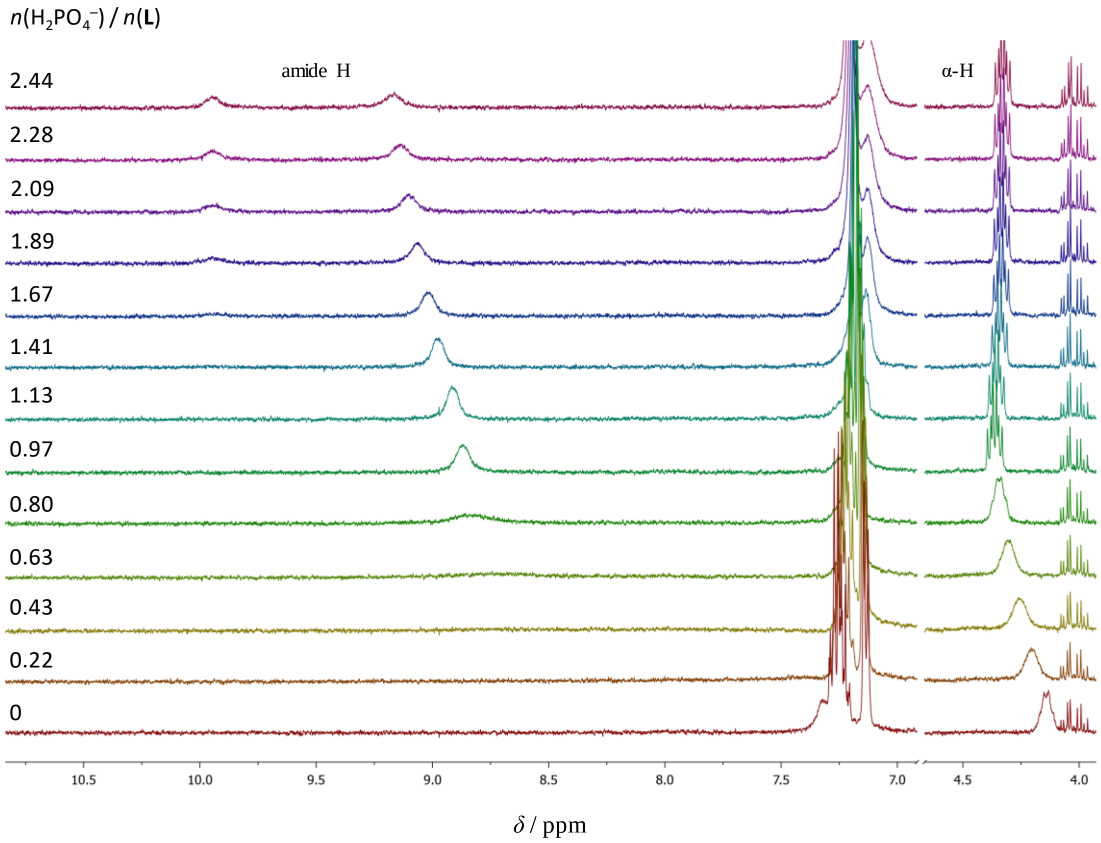

Figure S15. ^{1}H NMR titration of cyclopeptide **L** (c = 4.65 × 10^{-4} mol dm^{-3}) with $TBAH_2PO_4$ (c = 2.71 × 10^{-3} mol dm^{-3}) in deuterated acetonitrile.

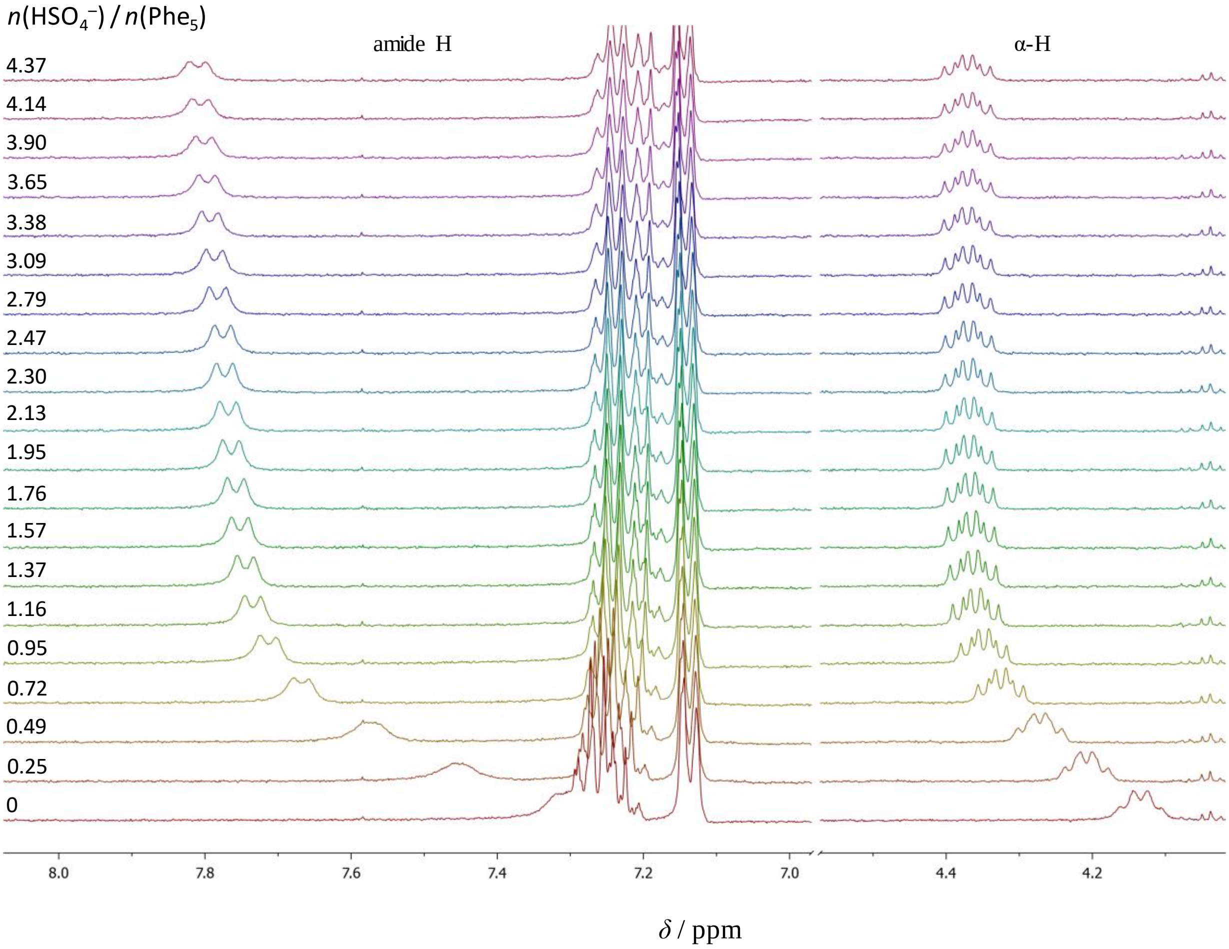

Figure S16. ^{1}H NMR titration of cyclopeptide **L** ($c = 4.19 \times 10^{-4}$ mol dm^{-3}) with TBAHSO$_4$ ($c = 5.34 \times 10^{-3}$ mol dm^{-3}) in deuterated acetonitrile.

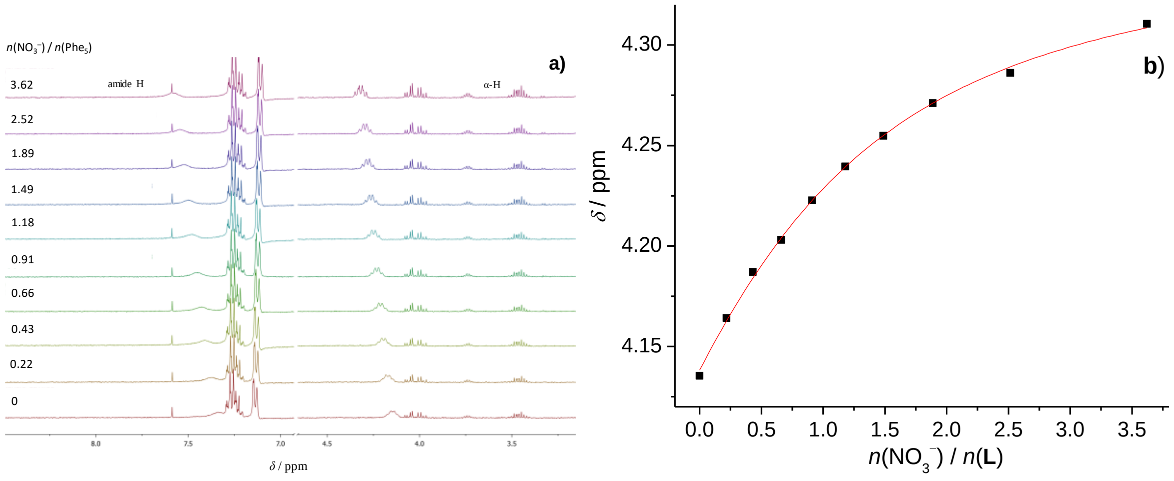

Figure S17. a) ^{1}H NMR titration of cyclopeptide **L** ($c = 4.26 \times 10^{-4}$ mol dm^{-3}) with TBANO$_3$ ($c = 2.67 \times 10^{-3}$ mol dm^{-3}) in deuterated acetonitrile. b) Dependence of the C-alpha proton chemical shift on molar ratio $n(NO_3^-)$ / n(**L**). ■ experimental; — calculated.

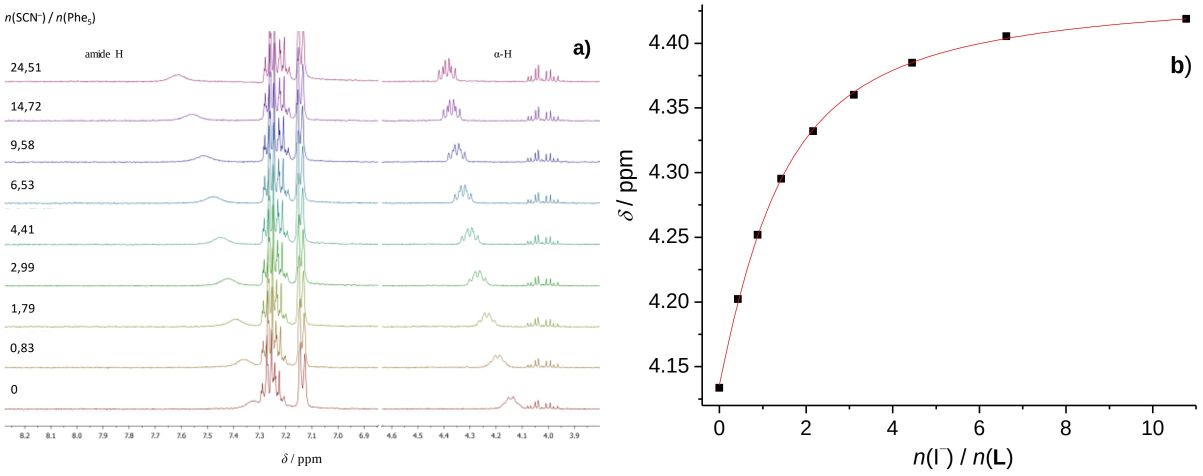

Figure S18. a) ^{1}H NMR titration of cyclopeptide **L** (c = 4.19 × 10^{-4} mol dm^{-3}) with TBASCN (c = 0.0178 mol dm^{-3}) in deuterated acetonitrile. b) Dependence of the C-alpha proton chemical shift on molar ratio n(I^{-}) / n(**L**). ■ experimental; — calculated.

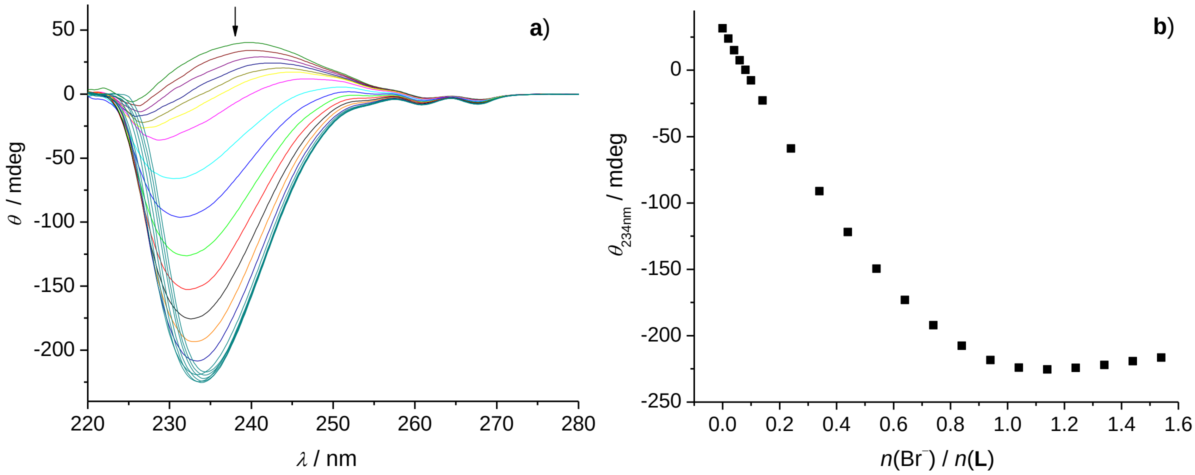

Figure S19. a) Circular dichroism titration of **L** (c = 5.0 × 10^{-4} mol dm^{-3}) with TBABr (c = 2.0 × 10^{-3} mol dm^{-3}) in acetonitrile at 25 °C. b) Dependence of circular dichroism on n(Cl^{-}) / n(**L**) molar ratio.

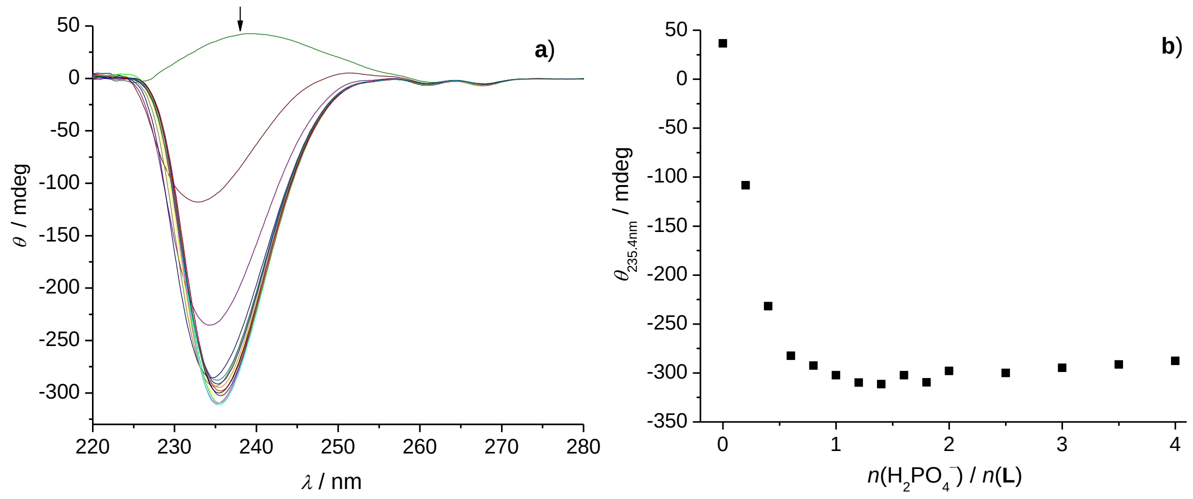

Figure S20. a) Circular dichroism titration of **L** (c = 5.0 × 10^{-4} mol dm^{-3}) with $TBAH_2PO_4$ (c = 0.01 mol dm^{-3}) in acetonitrile at 25 °C. b) Dependence of circular dichroism on $n(Cl^{-})$ / n(**L**) molar ratio.

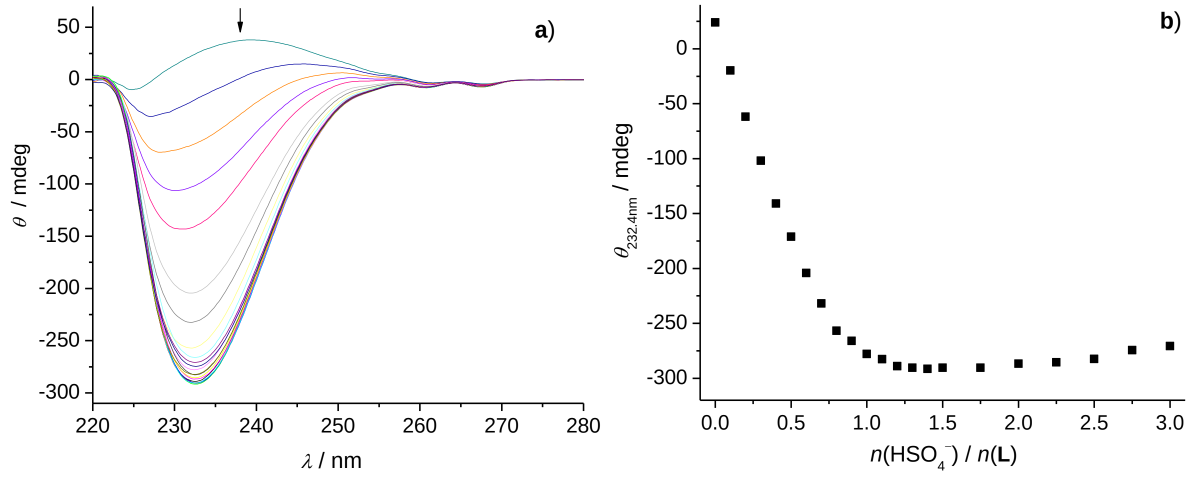

Figure S21. a) Circular dichroism titration of **L** (c = 5.0 × 10^{-4} mol dm^{-3}) with $TBAHSO_4$ (c = 5.0 × 10^{-3} mol dm^{-3}) in acetonitrile at 25 °C. b) Dependence of circular dichroism on $n(Cl^{-})$ / n(**L**) molar ratio.

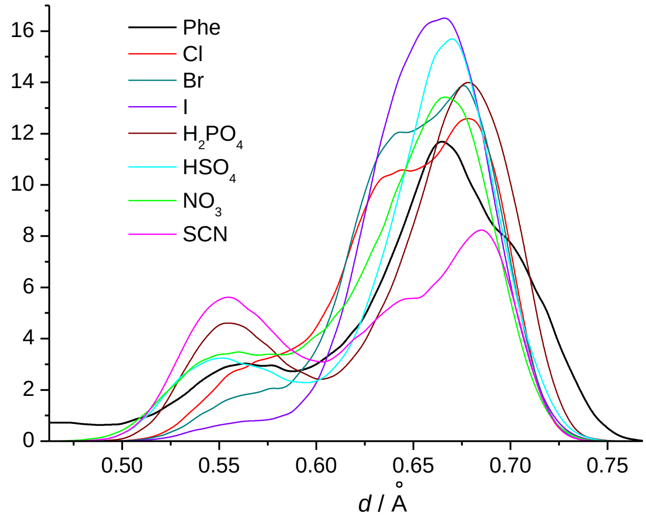

Figure S22. Distribution of center of mass of phenol rings and geometric center defined by C-alpha atoms of **L** anion complexes in acetonitrile.

MeOH

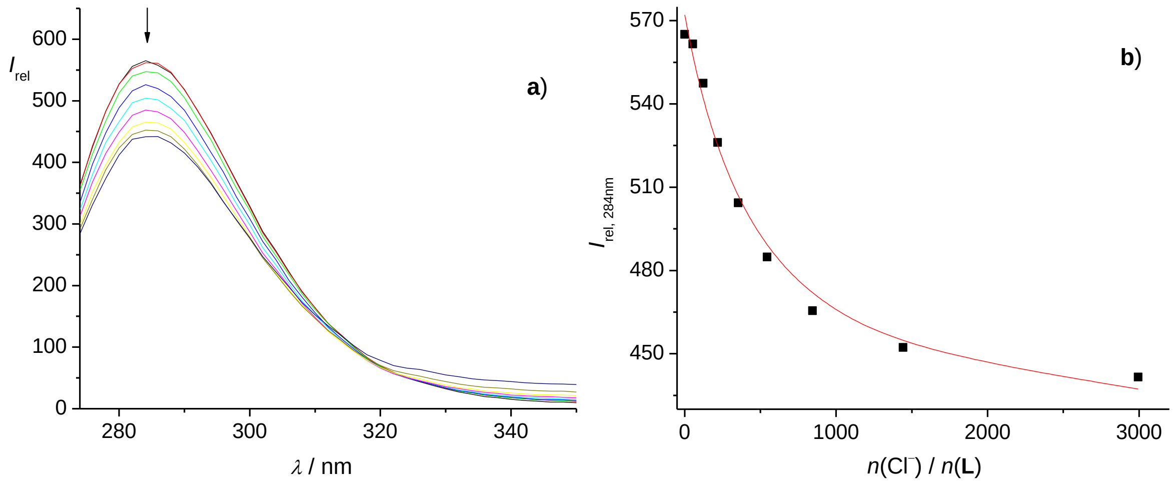

Figure S23. a) Fluorimetric titration of **L** (c = 5.10 × 10^{-5} mol dm^{-3}) with TEACl (c = 0.308 mol dm^{-3}) in methanol at 25 °C. b) Dependence of relative fluorescence intensity on $n(Cl^{-})$ / n(**L**) molar ratio. ■ experimental; — calculated.

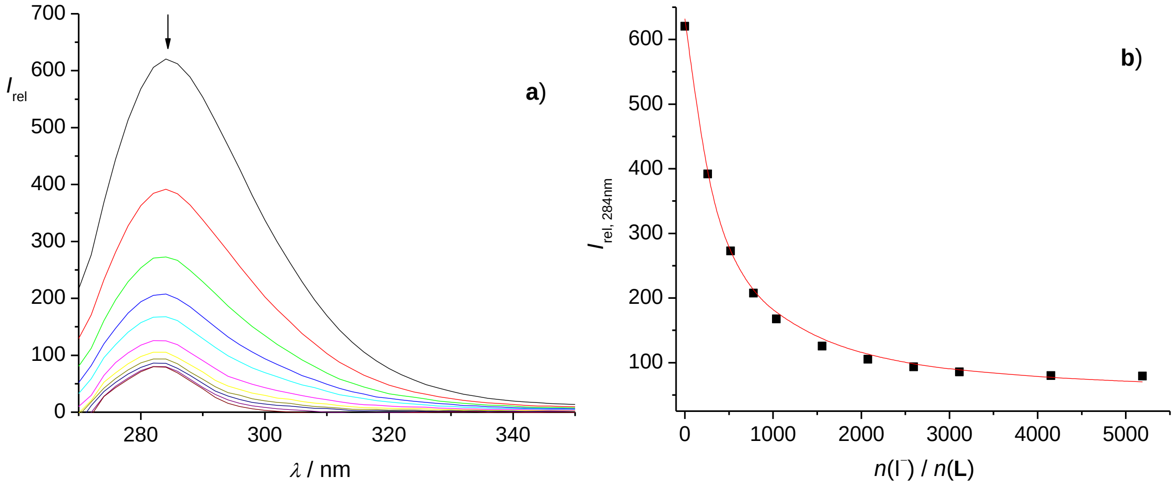

Figure S24. a) Fluorimetric titration of **L** (c = 5.22 × 10^{-5} mol dm^{-3}) with TBAI (c = 0.596 mol dm^{-3}) in methanol at 25 °C. b) Dependence of relative fluorescence intensity on $n(I^{-})$ / n(**L**) molar ratio. ■ experimental; — calculated.

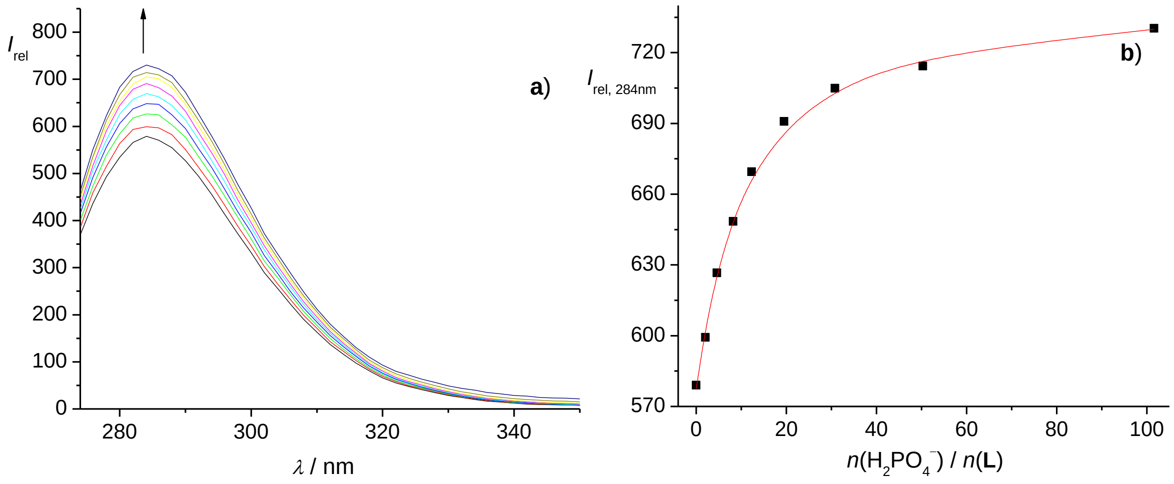

Figure S25. a) Fluorimetric titration of **L** (c = 5.09 × 10^{-5} mol dm^{-3}) with $TBAH_2PO_4$ (c = 0.012 mol dm^{-3}) in methanol at 25 °C. b) Dependence of relative fluorescence intensity on $n(H_2PO_4^-)$ / n(**L**) molar ratio. ■ experimental; — calculated.

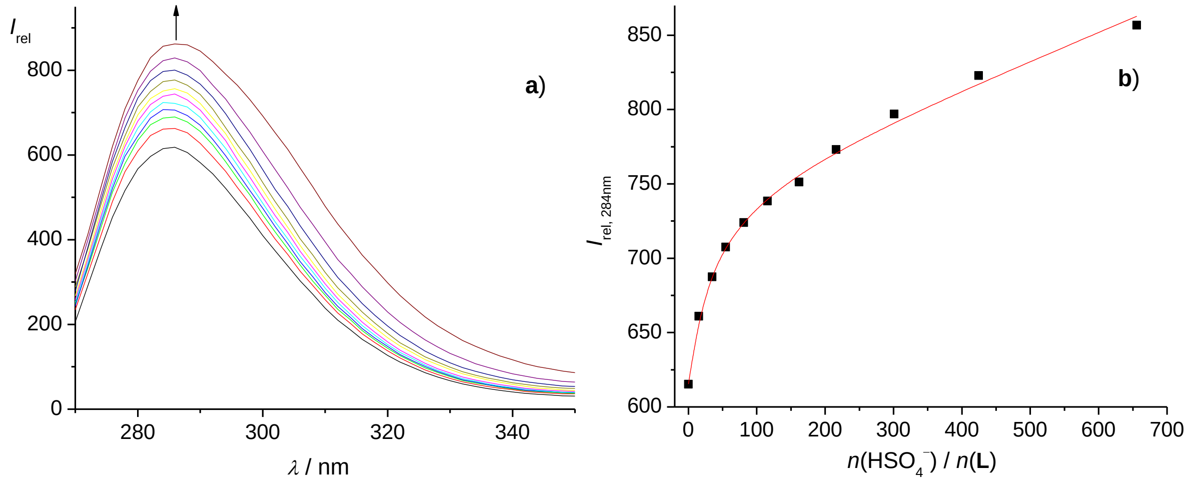

Figure S26. a) Fluorimetric titration of **L** (c = 5.09 × 10^{-5} mol dm^{-3}) with $TBAHSO_4$ (c = 0.0903 mol dm^{-3}) in methanol at 25 °C. b) Dependence of relative fluorescence intensity on $n(HSO_4^-)$ / n(**L**) molar ratio. ■ experimental; — calculated.

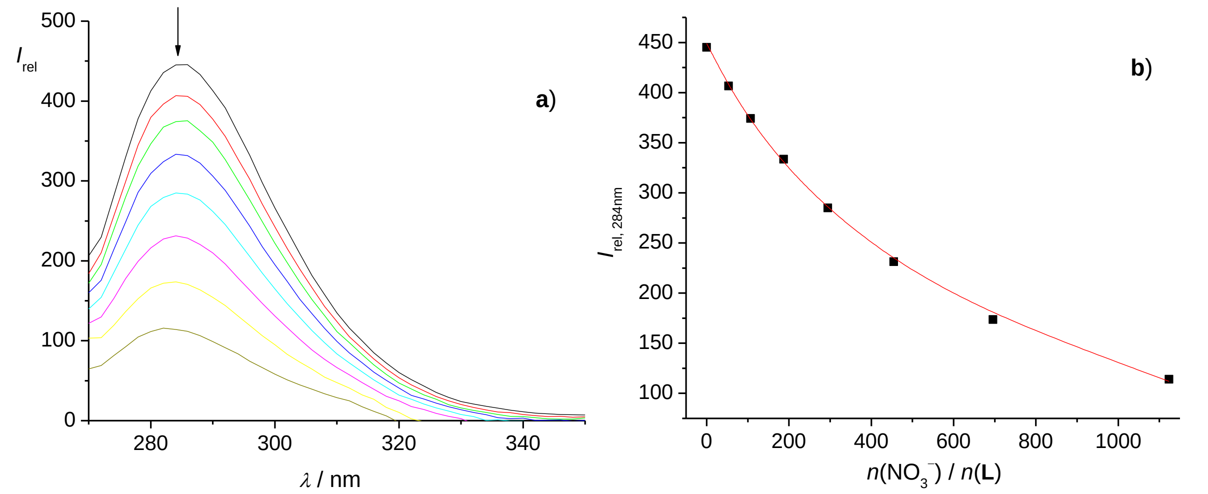

Figure S27. a) Fluorimetric titration of **L** (c = 5.10 × 10^{-5} mol dm^{-3}) with $TBANO_3^-$ (c = 0.60 mol dm^{-3}) in methanol at 25 °C. b) Dependence of relative fluorescence intensity on $n(NO_3^-)$ / n(**L**) molar ratio. ■ experimental; — calculated.

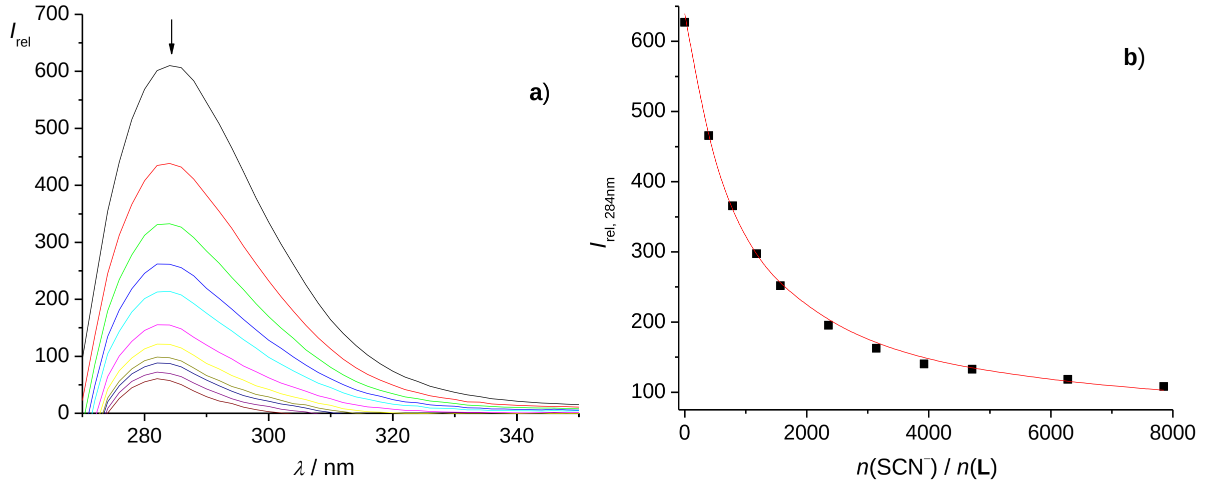

Figure S28. a) Fluorimetric titration of **L** (c = 5.22 × 10^{-5} mol dm^{-3}) with TBASCN (c = 0.901 mol dm^{-3}) in methanol at 25 °C. b) Dependence of relative fluorescence intensity on $n(Br^-)$ / n(**L**) molar ratio. ■ experimental; — calculated.

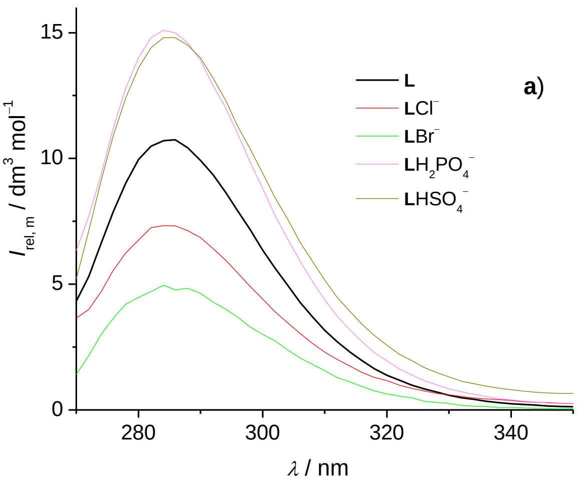

Figure S29. Molar fluorescence spectra of **L**-anion complexes in methanol at 25 °C. λ_{ex} = 260 nm, excitation slit = 10 nm, emission slit = 10 nm.

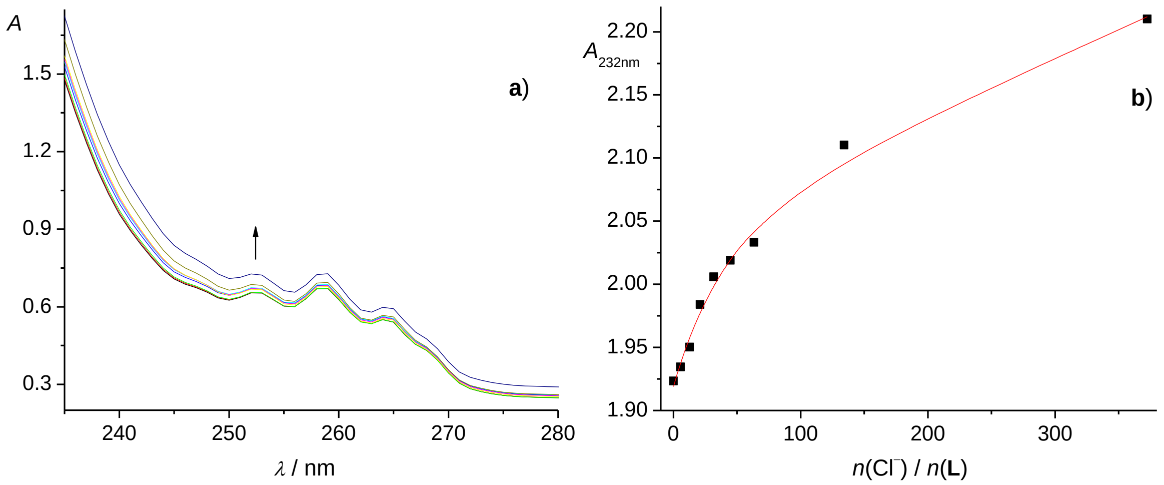

Figure S30. a) Spectrophotometric titration of **L** (c = 4.04 × 10^{-4} mol dm^{-3}) with TEACl (c = 0.301 mol dm^{-3}) in methanol at 25 °C. b) Dependence of absorbance on $n(Cl^-)$ / n(**L**) molar ratio. ■ experimental; — calculated.

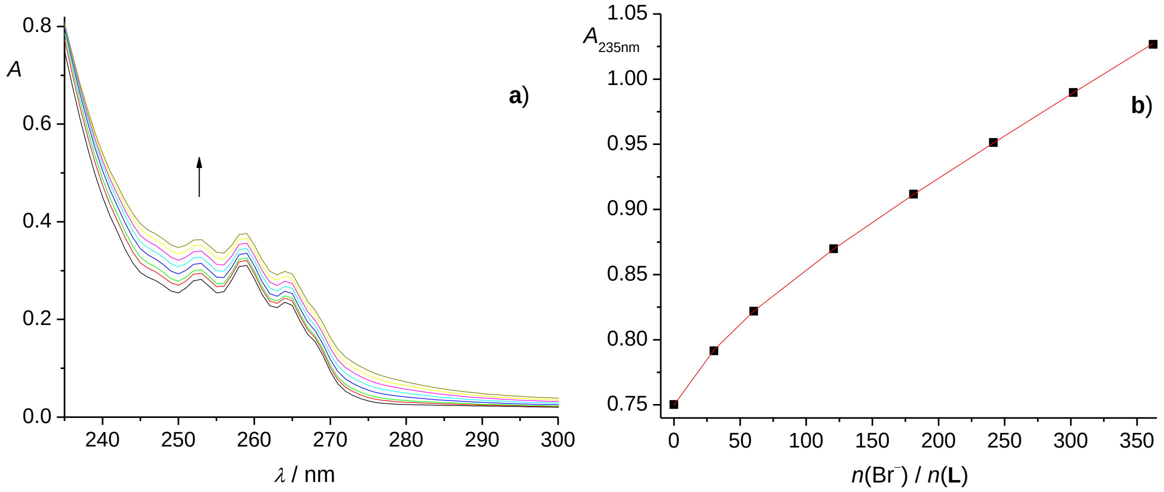

Figure S31. a) Spectrophotometric titration of **L** (c = 3.06 × 10^{-4} mol dm^{-3}) with TBABr (c = 0.406 mol dm^{-3}) in methanol at 25 °C. b) Dependence of absorbance on $n(Br^{-})$ / n(**L**) molar ratio. ■ experimental; — calculated.

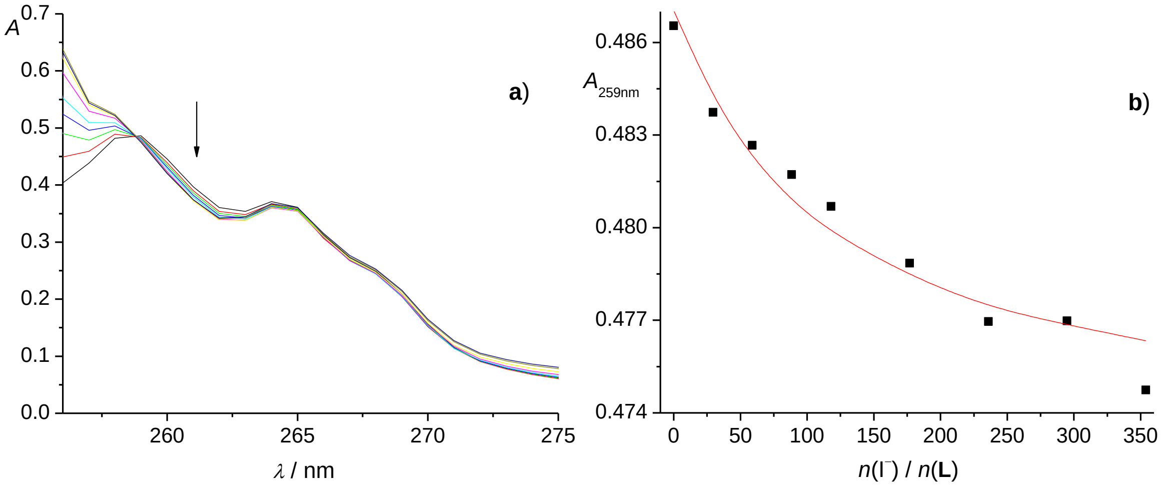

Figure S32. a) Spectrophotometric titration of **L** (c = 4.59 × 10^{-4} mol dm^{-3}) with TBAI (c = 0.596 mol dm^{-3}) in methanol at 25 °C. b) Dependence of apsorbance on $n(I^{-})$ / n(**L**) molar ratio. ■ experimental; — calculated.

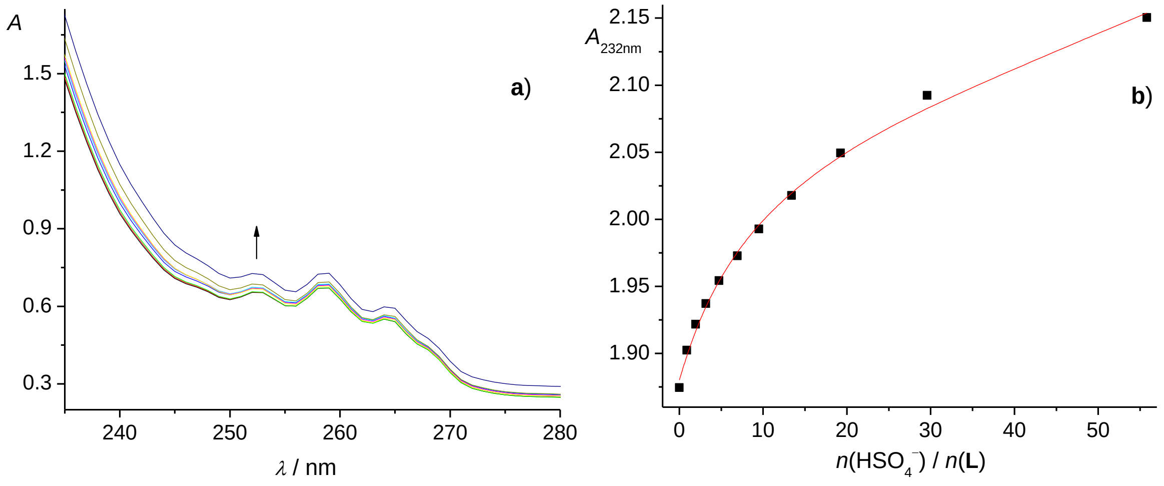

Figure S33. a) Spectrophotometric titration of **L** (c = 4.04 × 10^{-4} mol dm^{-3}) with TBAHSO$_4$ (c = 0.0451 mol dm^{-3}) in methanol at 25 °C. b) Dependence of apsorbance on n(HSO$_4^-$) / n(**L**) molar ratio. ■ experimental; — calculated.

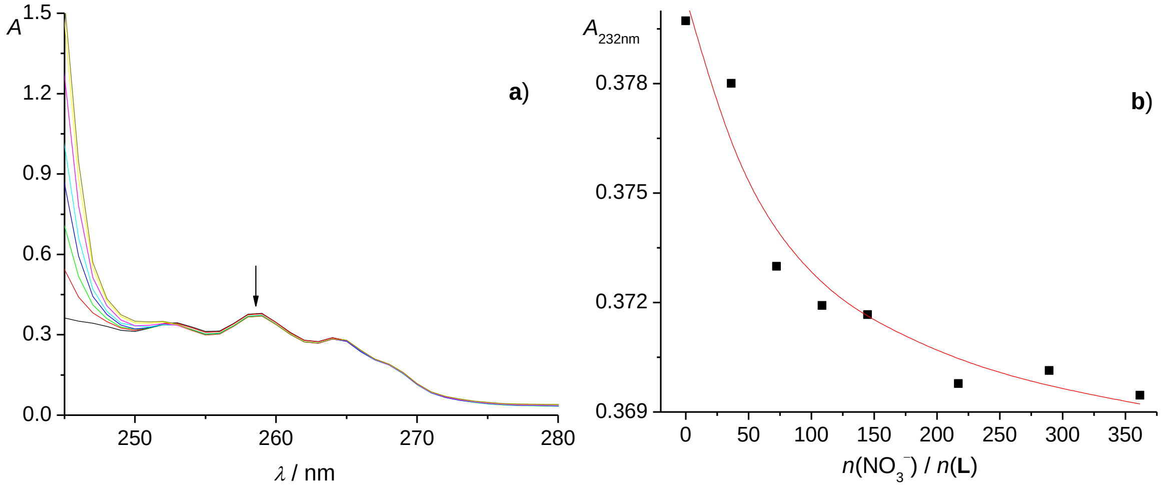

Figure S34. a) Spectrophotometric titration of **L** (c = 3.77 × 10^{-4} mol dm^{-3}) with TBANO$_3$ (c = 0.60 mol dm^{-3}) in methanol at 25 °C. b) Dependence of absorbance on n(NO$_3^-$) / n(**L**) molar ratio. ■ experimental; — calculated.

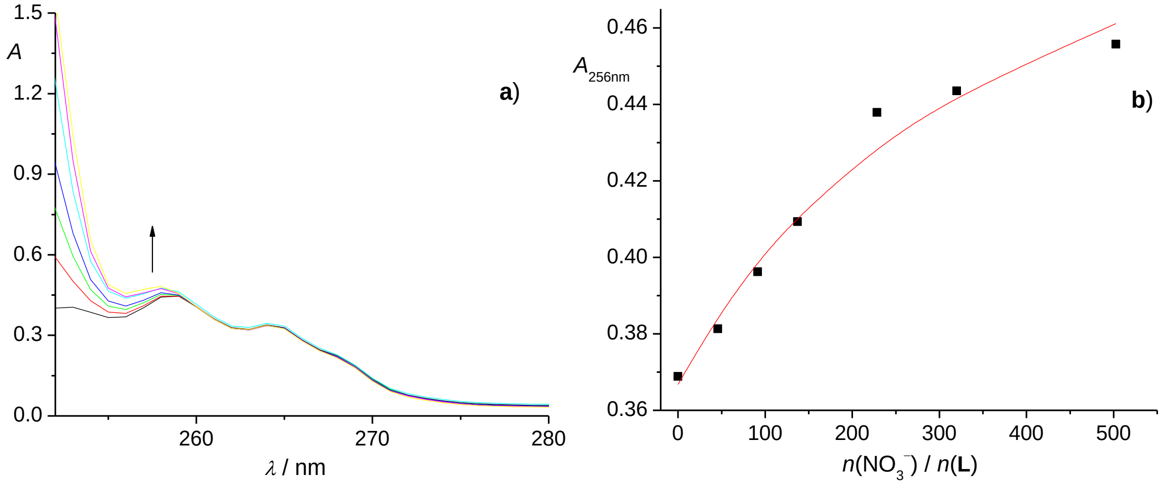

Figure S35. a) Spectrophotometric titration of **L** (c = 4.48 × 10^{-4} mol dm^{-3}) with TBASCN (c = 0.901 mol dm^{-3}) in methanol at 25 °C. b) Dependence of absorbance on $n(NO_3^{-})$ / n(**L**) molar ratio. ■ experimental; — calculated.

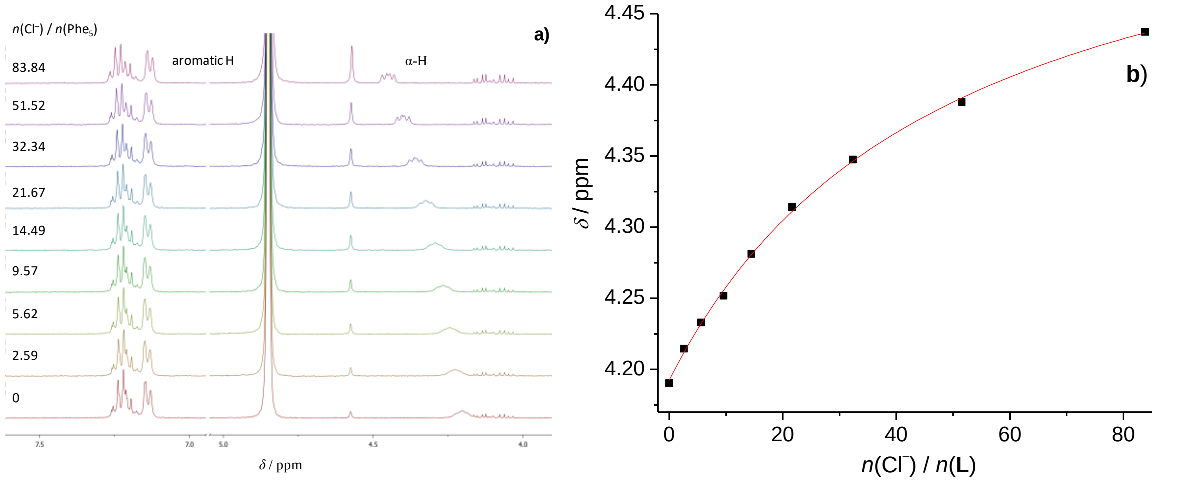

Figure S36. a) ^{1}H NMR titration of cyclopeptide **L** (c = 4.80 × 10^{-4} mol dm^{-3}) with TEACl (c = 0.0702 mol dm^{-3}) in deuterated methanol. b) Dependence of the C-alpha proton chemical shift on molar ratio $n(Cl^{-})$ / n(**L**). ■ experimental; — calculated.

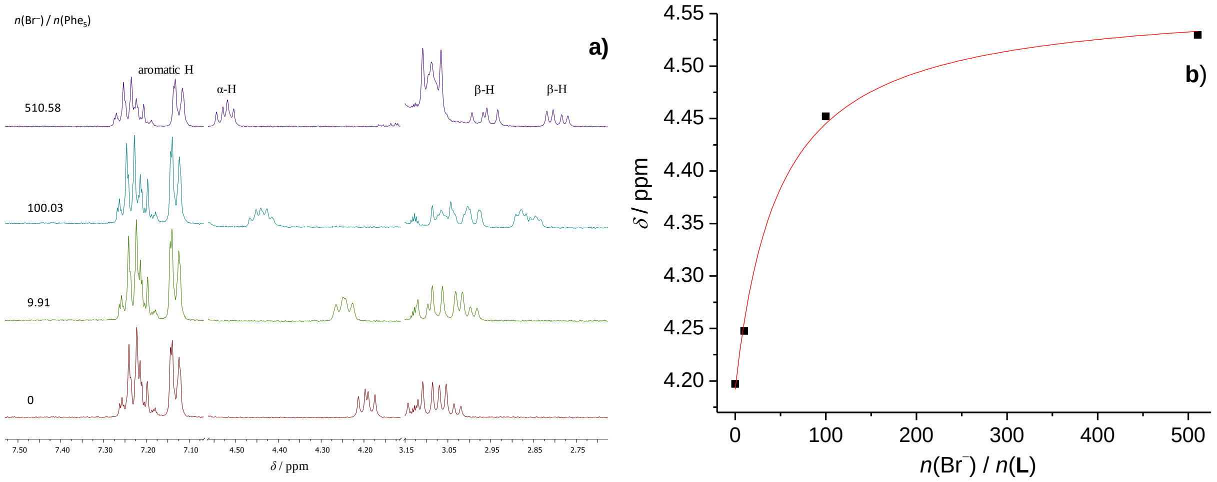

Figure S37. a) ^{1}H NMR titration of cyclopeptide **L** (c = 5.44 × 10^{-4} mol dm^{-3}) with TBABr (c = 0.555 mol dm^{-3}) in deuterated methanol. b) Dependence of the C-alpha proton chemical shift on molar ratio n(Br^{-}) / n(**L**). ■ experimental; — calculated.

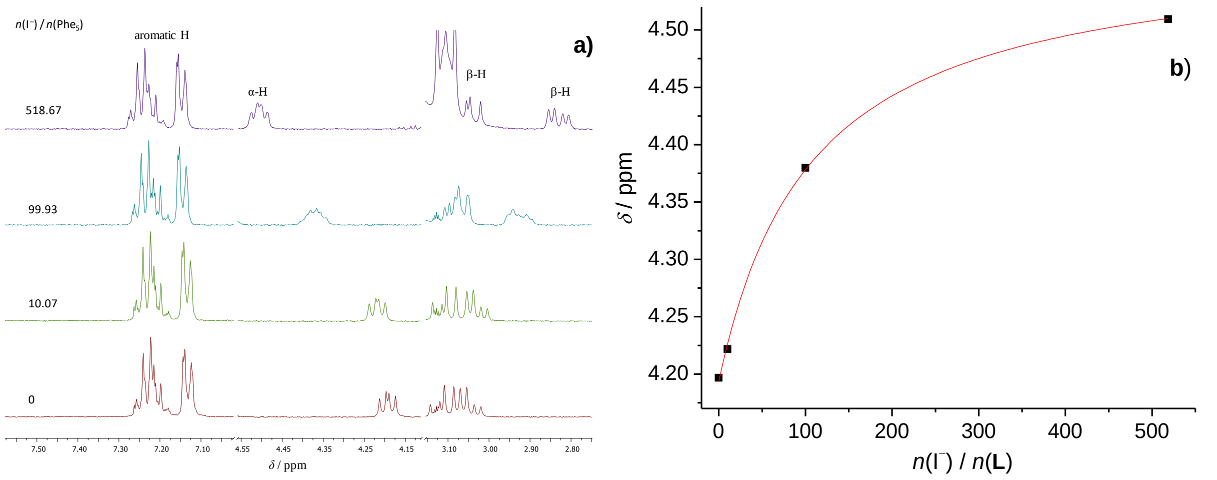

Figure S38. a) ^{1}H NMR titration of cyclopeptide **L** (c = 5.44 × 10^{-4} mol dm^{-3}) with TBAI (c = 0.564 mol dm^{-3}) in deuterated methanol. b) Dependence of the C-alpha proton chemical shift on molar ratio n(I^{-}) / n(**L**). ■ experimental; — calculated.

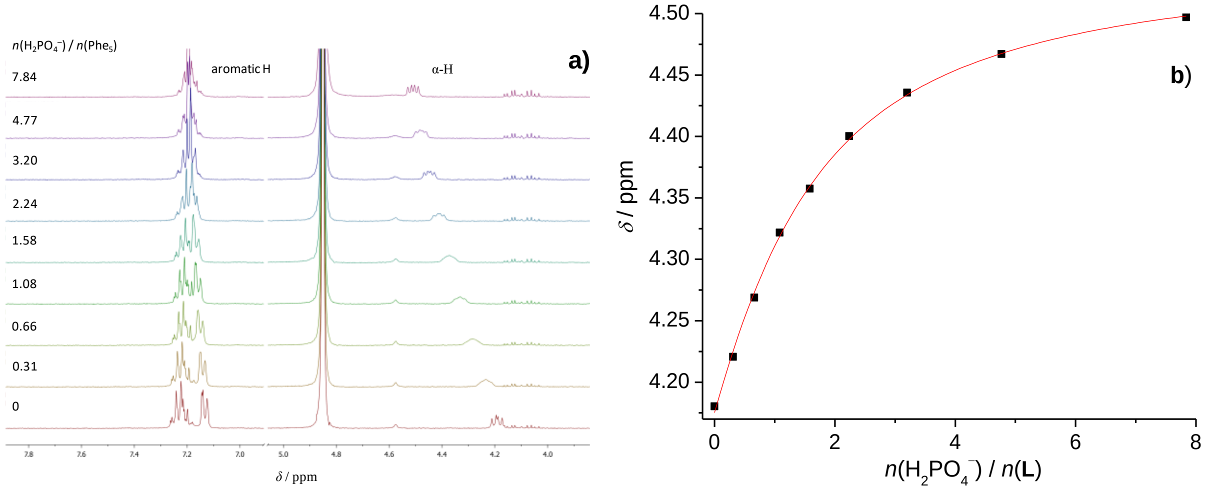

Figure S39. a) ^{1}H NMR titration of cyclopeptide **L** (c = 4.19 × 10^{-4} mol dm^{-3}) with TBAH_2PO_4 (c = 6.58 × 10^{-3} mol dm^{-3}) in deuterated methanol. b) Dependence of the C-alpha proton chemical shift on molar ratio $n(H_2PO_4^-)$ / n(**L**). ■ experimental; — calculated.

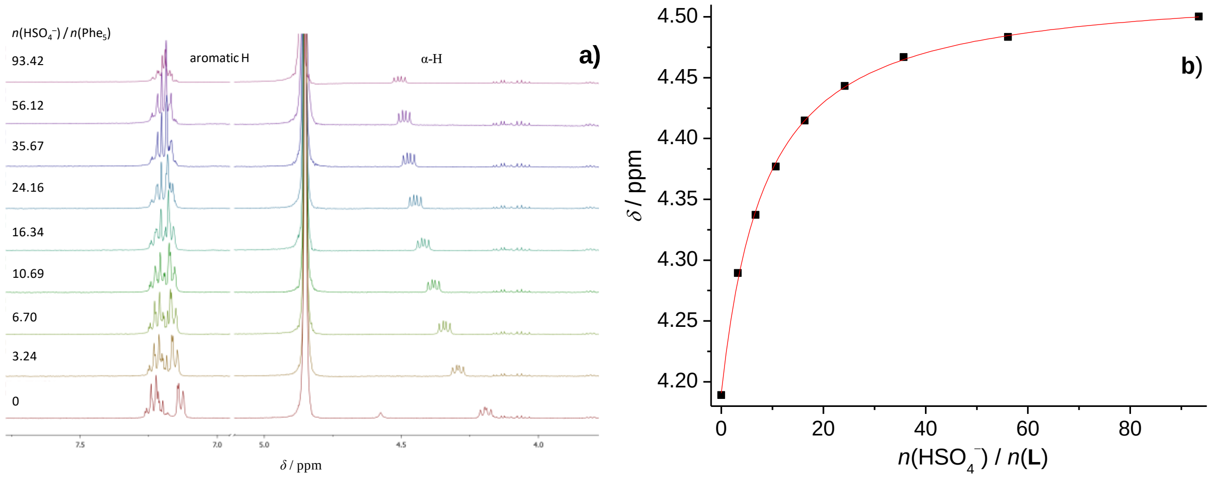

Figure S40. a) ^{1}H NMR titration of cyclopeptide **L** (c = 4.42 × 10^{-4} mol dm^{-3}) with TBAHSO_4 (c = 8.09 × 10^{-2} mol dm^{-3}) in deuterated methanol. b) Dependence of the C-alpha proton chemical shift on molar ratio $n(HSO_4^-)$ / n(**L**). ■ experimental; — calculated.

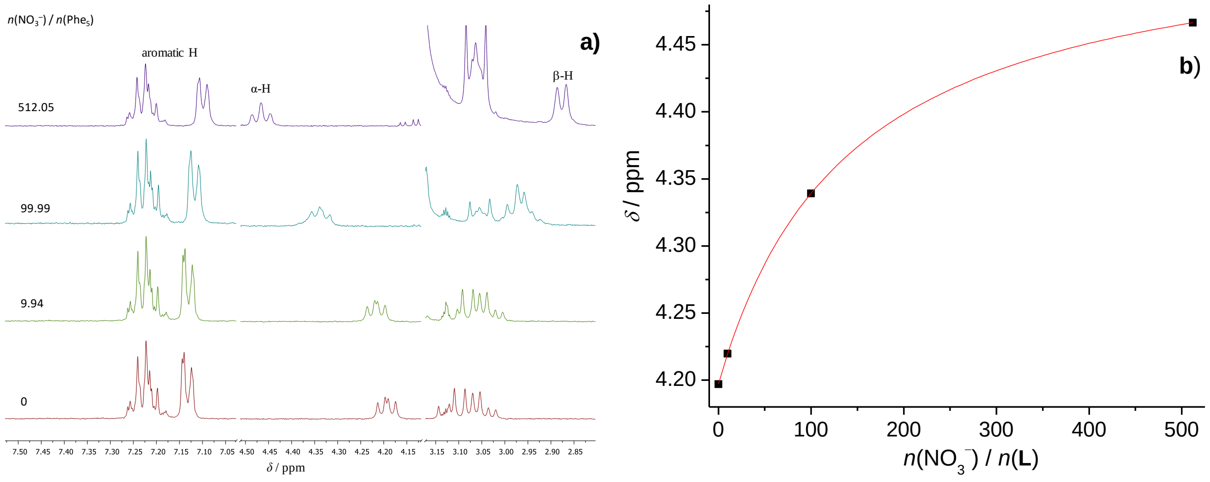

Figure S41. a) ^{1}H NMR titration of cyclopeptide **L** (c = 5.44 × 10^{-4} mol dm^{-3}) with TBANO3 (c = 0.557 mol dm^{-3}) in deuterated methanol. b) Dependence of the C-alpha proton chemical shift on molar ratio $n(NO_3^-)$ / n(**L**). ■ experimental; — calculated.

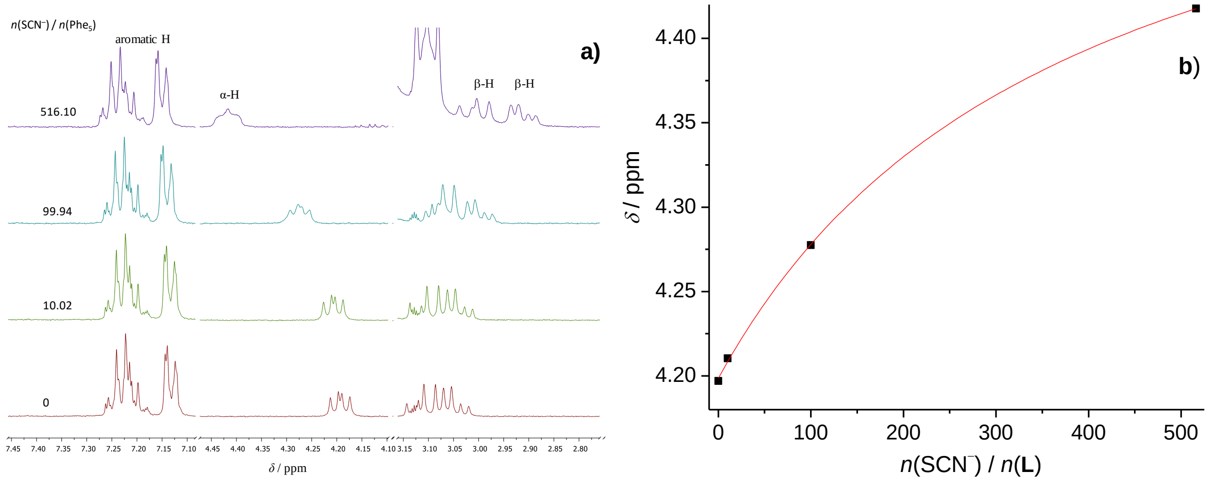

Figure S42. a) ^{1}H NMR titration of cyclopeptide **L** (c = 5.44 × 10^{-4} mol dm^{-3}) with TBASCN (c = 0.561 mol dm^{-3}) in deuterated methanol. b) Dependence of the C-alpha proton chemical shift on molar ratio $n(SCN^-)$ / n(**L**). ■ experimental; — calculated.

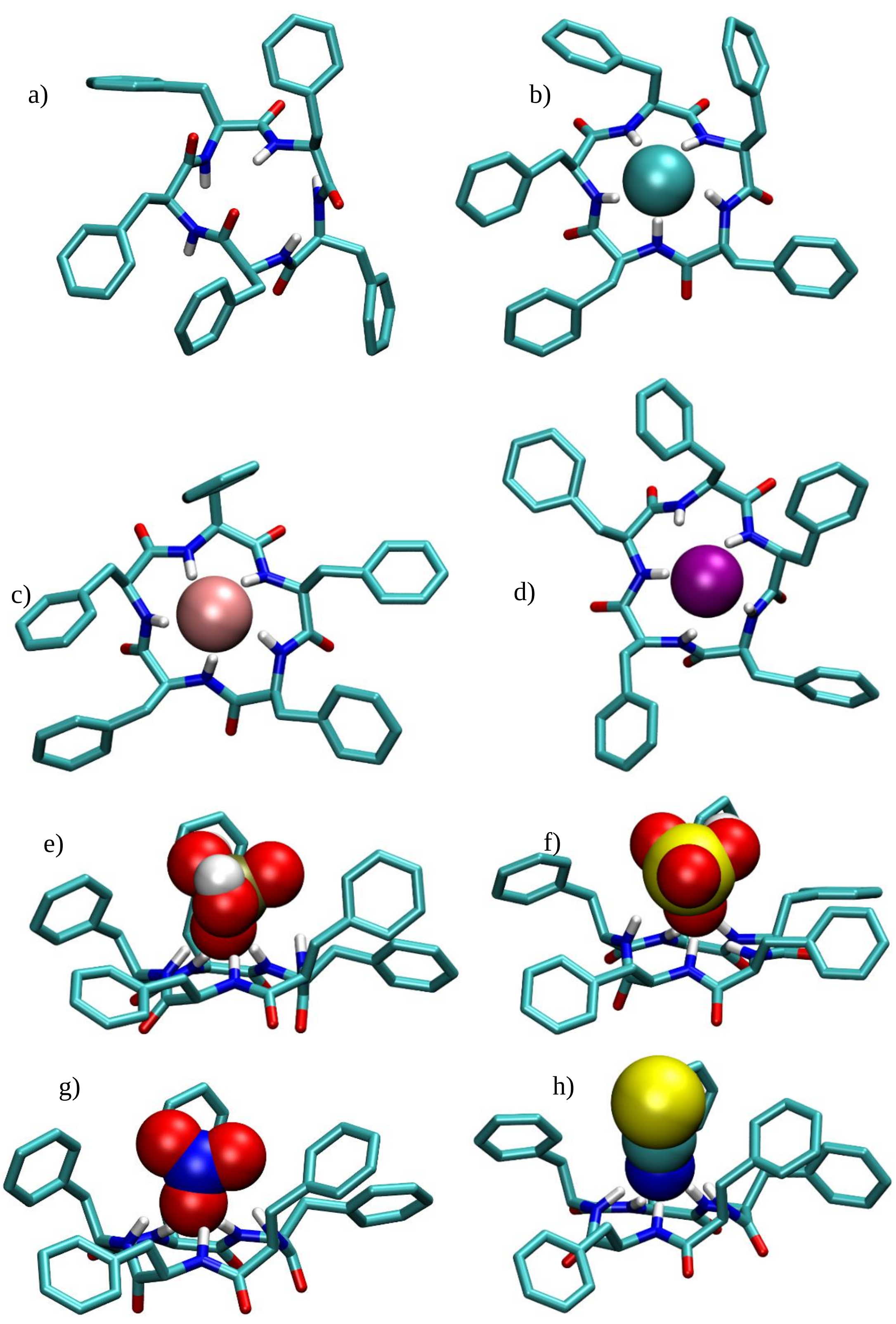

Figure S43. Structures of a) free **L** and anion complexes of **L** with b) chloride, c) bromide, d) iodide, e) dihydrogen phosphate, f) hydrogen sulfate, g) nitrate and h) thiocyanate in methanol obtained by molecular dynamics simulations. Hydrogen atoms bound to carbon atoms of **L** are omitted for clarity.

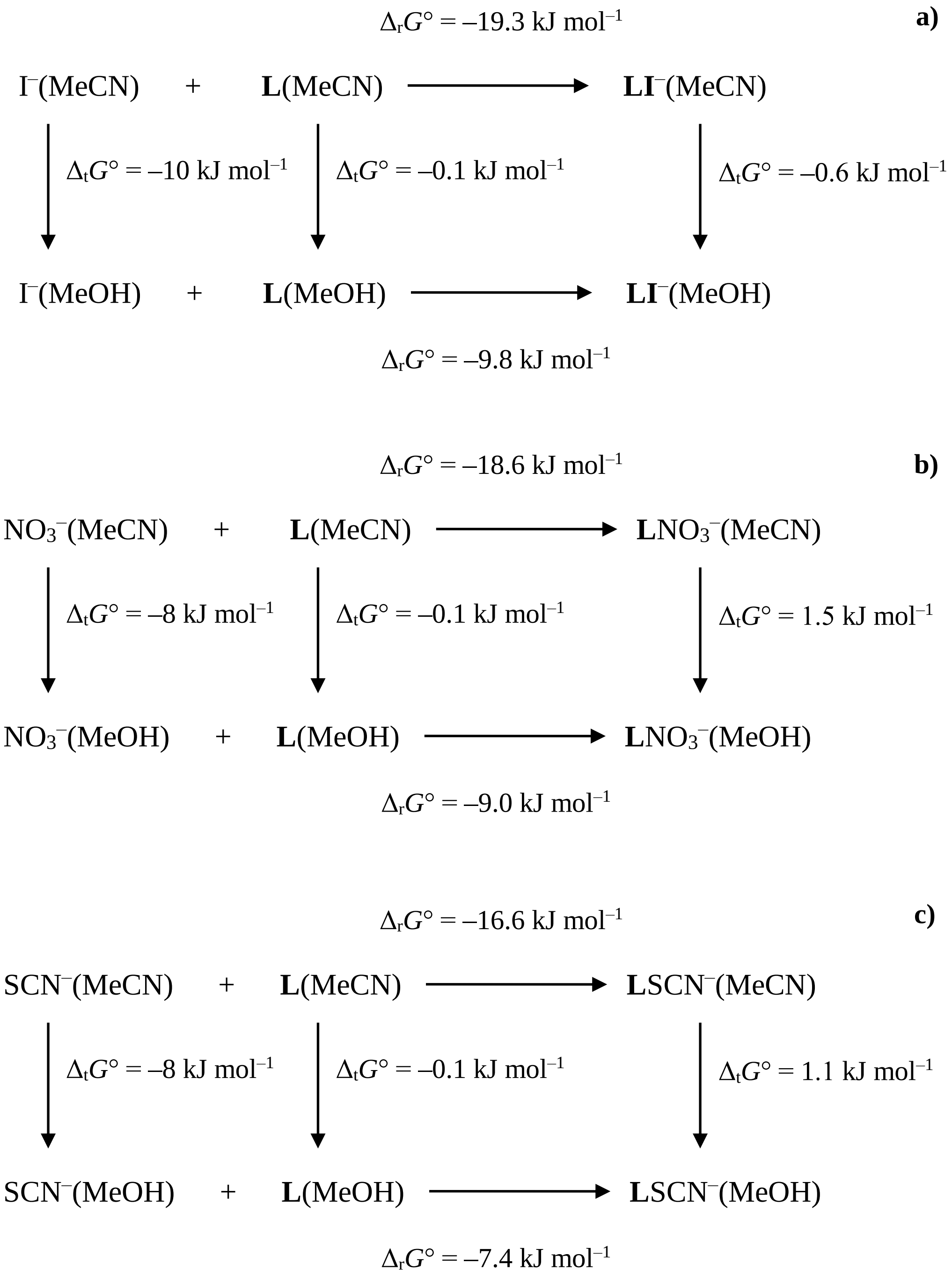

Scheme S1. Thermodynamic cycles for complexation of a) I^-, b) NO_3^- and c) SCN^- with **L** in acetonitrile and methanol expressed in terms of Gibbs energies.
